# Environmental stress promotes the persistence of facultative bacterial symbionts in amoebae

**DOI:** 10.1002/ece3.9899

**Published:** 2023-03-16

**Authors:** Zihe Wang, Wei Huang, Yingwen Mai, Yuehui Tian, Bo Wu, Cheng Wang, Qingyun Yan, Zhili He, Longfei Shu

**Affiliations:** ^1^ School of Environmental Science and Engineering, Southern Marine Science and Engineering Guangdong Laboratory (Zhuhai), Guangdong Provincial Key Laboratory of Environmental Pollution Control and Remediation Technology, State Key Laboratory for Biocontrol Sun Yat‐sen University Guangzhou China

**Keywords:** amoeba, amoeba‐resisting bacteria, environmental stress, pollution, protist, symbiosis

## Abstract

Amoebae are one major group of protists that are widely found in natural and engineered environments. They are a significant threat to human health not only because many of them are pathogenic but also due to their unique role as an environmental shelter for pathogens. However, one unsolved issue in the amoeba–bacteria relationship is why so many bacteria live within amoeba hosts while they can also live independently in the environments. By using a facultative amoeba– *Paraburkholderia* bacteria system, this study shows that facultative bacteria have higher survival rates within amoebae under various environmental stressors. In addition, bacteria survive longer within the amoeba spore than in free living. This study demonstrates that environmental stress can promote the persistence of facultative bacterial symbionts in amoebae. Furthermore, environmental stress may potentially select and produce more amoeba‐resisting bacteria, which may increase the biosafety risk related to amoebae and their intracellular bacteria.

## Graphical abstract

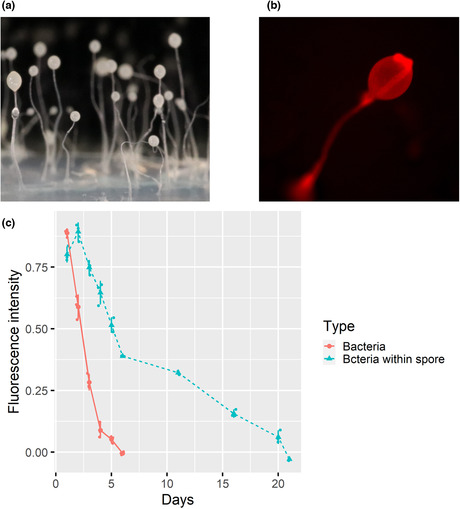

## 1 INTRODUCTION

Amoebae are single‐celled protists that are widely found in water, soil, and other natural habitats (Samba‐Louaka et al., 2019; Zheng et al., 2022). They have also been found in engineered systems such as swimming pools and drinking water systems (Delafont et al., 2013; Thomas et al., 2006; Thomas & Ashbolt, 2011). Amoebae are a significant threat to human health because some of them are pathogenic and even lethal to humans (Shi et al., 2021; Thomas & Ashbolt, 2011). In addition, diverse microbes, including bacteria, fungi, and viruses, many of which are human pathogens, have been found hidden within amoebae (Shi et al., 2021; Strassmann & Shu, 2017). For example, *Legionella pneumophila* can reside within amoebae, using them as their replication hosts (Hoffmann et al., 2014). Moreover, recent studies suggest that amoebae are resistant to drinking water disinfection and further protect their intracellular bacteria from disinfection (He et al., 2021, 2022). Therefore, both amoebae and their bacterial symbionts are a significant threat to public health, and a better understanding of their interactions is in urgent need.

One unsolved issue in the amoeba–bacteria relationship is why so many facultative symbionts live within amoeba hosts while they can also live independently in the environments. Host–symbiont interactions are prevalent in nature and can significantly impact each other's fitness (Husnik et al., 2021; Shi et al., 2021; Shu, Brock, et al., 2018). One benefit of symbiotic relationships is improving their adaptability and resistance to stressful environments. For instance, the metabolites of endosymbiotic dinoflagellates can neutralize oxidative damage and protect corals from exposure to intense ultraviolet radiation and high temperatures (Putnam et al., 2012). The symbiotic algae can increase green polyps' thermal resistance (Ye et al., 2019). Salt and alkali tolerance in plants such as *Arabidopsis thaliana* can be enhanced by the symbiotic fungus, whose ATPases can reduce the sodium content of plant cells (Metzger et al., 2019). Obligate symbiotic partners can be mutually beneficial to amoebae, and they were found to be protective against bacterial and viral pathogens (Arthofer et al., 2022; Lee et al., 2019; Sakuragi et al., 2010). Although obligate symbionts share common interests with their hosts, facultative symbionts are often acquired from the environment and can live independently. Therefore, compared with living in nutrition‐rich environments, it is not clear why many bacteria live within amoeba, which raises the question: why do so many facultative symbionts live within amoeba hosts? Do they really benefit from their amoeba hosts?

However, it is challenging to address these questions because most current studies focus on the host's fitness consequences, while the host's effect on facultative symbionts remains largely unknown due to the difficulty of measuring symbiont fitness (Garcia et al., 2019; Garcia & Gerardo, 2014). Therefore, a better understanding of the fitness of facultative symbionts can help explain why facultative symbionts tend to live with amoeba hosts and how host–microbe relationships persist. The social amoeba–*Paraburkholderia* symbioses provide a trackable system to address this question. Three *Paraburkholderia* symbionts (*P. agricolaris*, *P. hayleyella*, and *P. bonniae*) can initiate a stable association with naive *D. discoideum* hosts (Brock et al., 2020). They are mostly inedible, but they benefit the amoebae by inducing the secondary carriage of food bacteria (Brock et al., 2011; DiSalvo et al., 2015; Shu, Brock, et al., 2018). The *Paraburkholderia* presumably benefit by living inside the amoebae. However, one study showed that *D. discoideum* inhibited the growth rate of both *Paraburkholderia* species, and only *P. hayleyella* benefited under some conditions (Garcia et al., 2019). Another study showed that *Paraburkholderia* could reduce intraspecific competition after being dispersed by the host to a food‐poor environment (Scott et al., 2022). However, it is still unclear whether these *Paraburkholderia* benefit from living inside the amoebae.

**FIGURE 1:**
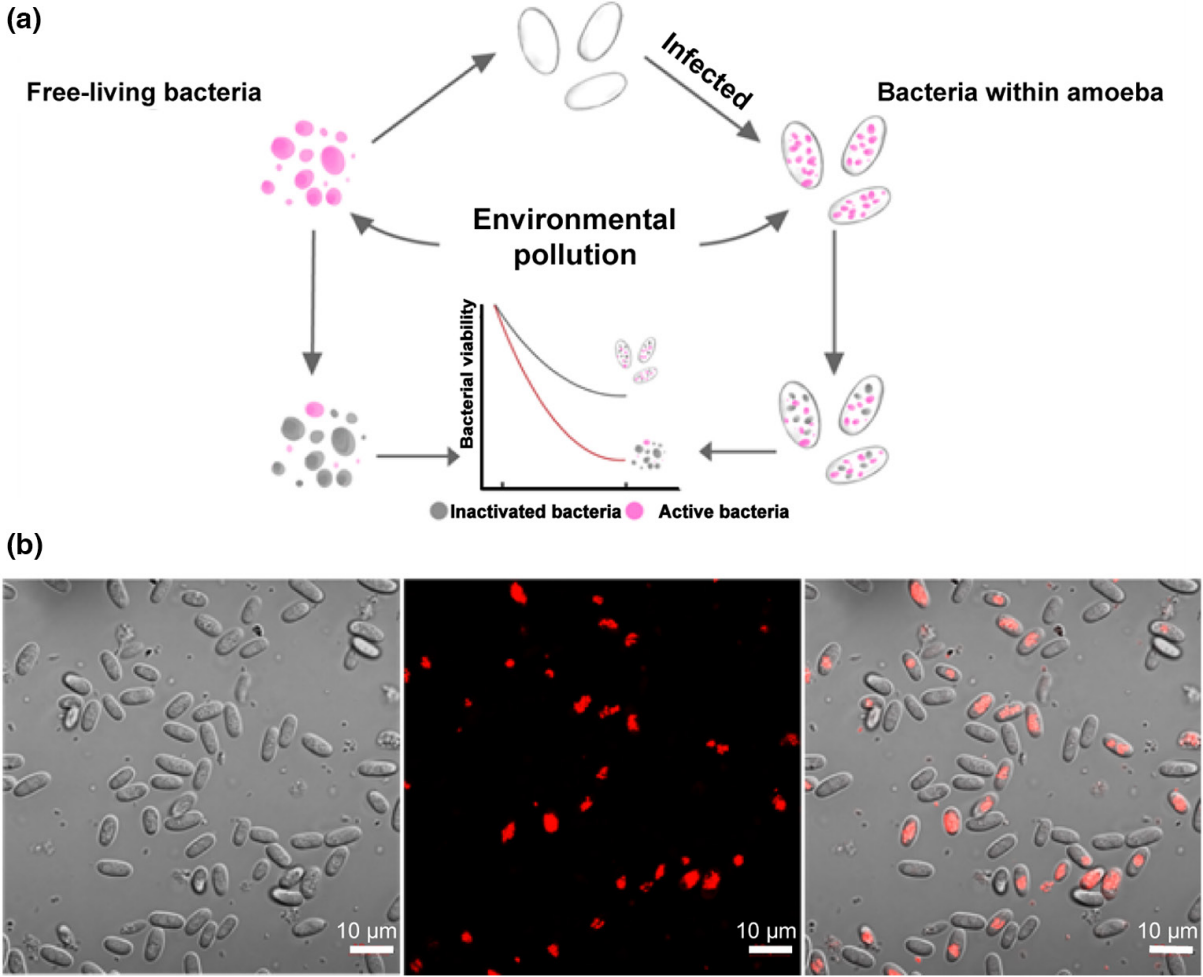
(a) Schematic diagram of the experimental design; (b) construction of the facultative amoeba–bacteria system. Intracellular bacteria are labeled with the red fluorescent label (RFP).

Environmental pollutants, such as heavy metals, acid rain, and greenhouse gas, are a key challenge for life on Earth, affecting the growth and survival of organisms and acting as a strong selective force (Whitehead et al., 2017). Amoebae, an influential group of protists, can have complex relationships with environmental stressors (Wang et al., 2023; Wu et al., 2022; Yu et al., 2022; Zhang et al., 2022). In this study, we hypothesized that amoebae could protect their facultative bacterial symbionts from various environmental pollutants, promoting the persistence of facultative bacterial symbionts against environmental stress. We obtained a facultative amoeba–bacteria system and treated them with various environmental pollutants (Figure 1a). Then, we quantitatively measure the fitness of facultative symbionts and evaluate the protective role of amoebae, aiming to explain how amoeba–bacterium relationships form and persist at environmental time scales.

## 2 MATERIALS AND METHODS

### 2.1 Cultivation of amoebae

This study used a model amoeba *Dictyostelium discoideum* QS9 for all experiments (Tian et al., 2022; Zhang et al., 2022). *D. discoideum* was grown from previously frozen spores on SM/5 agar plates (2 g glucose, 2 g BactoPeptone (Oxoid), 2 g yeast extract (Oxoid), 0.2 g MgCl_2_, 1.9 g KH_2_PO_4_, 1 g K_2_HPO_4_, and 15 g agar per liter) with food bacterium *Klebsiella pneumoniae* (obtained from the Dicty Stock Center) at 21°C.

### 2.2 Acquisition of the facultative amoeba–bacteria system

We used a *Paraburkholderia* bacterium *P. agricolaris* B1qs70, known to be capable of surviving within amoebae or living by themselves, to acquire the facultative amoeba–bacteria system (Shu, Brock, et al., 2018; Shu, Zhang, et al., 2018). We used *Dictyostelium discoideum* QS9 because it was a non‐farmer strain. Therefore, the effects observed in this study were more focused on environmental scales. We grew RFP‐labeled *P. agricolaris* B1qs70 on SM/5 agar plates, collected stationary phase bacteria with inoculating loop, and suspended them in KK2 buffer (2.2 g KH_2_PO_4_ monobasic, 0.7 g K_2_HPO_4_ dibasic per liter). The bacterial suspension was set to an OD_600_ of 1.5 and was used for the following experiment. To set up the system, we plated 2 × 10^5^ amoeba spores with a mixture of *P. agricolaris* B1qs70 and *K. pneumoniae* (5%: 95% vol) on SM/5 plates at 21°C. After 5 days, amoeba spores are collected with KK2 buffer in 2‐mL falcon tubes by using an inoculation loop and counted on a hemocytometer using a light microscope.

Confocal microscopy was used to confirm the presence of bacteria in amoeba spores (Figure 1b). Spores were collected from the fruiting bodies into KK2 + 1% calcofluor white and imaged directly using Nikon A1Si Laser Scanning confocal microscope and Nikon Elements software. RFP‐labeled *P. agricolaris* B1qs70 was excited using the 561 laser and Calcoflour‐white with the 408 laser.

### 2.3 Exposure experiments of the amoeba–bacteria system to environmental stressors

Six environmental stressors were chosen to investigate how they would affect the symbiont's fitness. We have performed preliminary assays to choose the experimental conditions for the test, in which >99% of free‐living *P. agricolaris* B1qs70 could be inactivated after environmental stress treatment (Figure S1). For the exposure experiments, 1 mL of free‐living *P. agricolaris* B1qs70 (10^8^ cells) and 1 mL of amoeba spores (10^7^ cells) that contain *P. agricolaris* B1qs70 (around 10^8^ cells) were treated under heat stress (55°C for 20 min), heavy metal stress (200 mg/L Cu^2+^ for 20 min), acid stress (pH 1.0 HCl for 20 min), alkali stress (pH 12.0 NaOH for 20 min), salt stress (350 g/L NaCl for 20 min), and UV stress (ultraviolet lamp, 400 nm, 2.95 mW/m^2^ for 2 min), respectively. After the treatment, free‐living *P. agricolaris* B1qs70 and amoeba spores were washed four times with KK2 buffer and collected for the following analyses.

### 2.4 Quantification of symbiont's fitness

The survival of free‐living bacteria and facultative bacteria was defined as a logarithmic reduction (N/N0), where N0 and N were the numbers of viable cells before and after treatments. The control represents the number of viable bacteria (free‐living and intracellular) without stress, while the treatment represents the number of viable bacteria (free‐living and intracellular) after exposure to different environmental stressors. *P. agricolaris* B1qs70 are counted using the colony‐forming unit (CFU) method. Free‐living bacteria were directly counted through serial dilution and CFU counting. For intracellular symbionts, they were first released from amoeba spores using a homemade grind bead‐based method. Amoeba spores were mixed with 0.3 mL ZR BashinaBead (0.5 and 0.1 mm diameters) in a 2.0‐mL centrifuge tube, which were then broken on a FastPrep‐24 5 G high‐speed pyrolysis biological sample table homogenizer four times. The oscillation speed is 4.5 M/s for discontinuous intermittent oscillation (40 s shock + 5 min cooling) (He et al., 2021). Microscopic observation was used to confirm that all amoeba spores were broken and released intracellular bacteria, which were then counted using the CFU method on SM/5 plate at 25°C.

### 2.5 Morphology and survival of amoeba spores

The integrity and morphological changes of host amoeba spores under different environmental stresses were recorded by confocal microscopy. Subsequently, to test the viability of amoeba spores after treatments, amoeba spores (2 × 10^5^) were collected and cultured with 200 μL *K. pneumoniae* suspension (OD_600_ = 1.5) on SM/5 medium at 21°C. The plates were checked every 24 h for 4–5 days.

### 2.6 Maximum survival time of free‐living and amoeba‐hosted bacteria

We also compared the maximum survival time of free‐living bacteria versus intracellular bacteria within amoeba (Figure 4a) to investigate whether living within amoeba hosts gave them a fitness advantage. B1Qs70 strain labeled with RFP was constructed through triparental mating technique along with *E. coli* helper strain E1354 (pTNS3‐asdEc) and the *E. coli* donor strain E2072 with pmini‐Tn7‐gat‐P1‐rfp as previously described (Shu, Brock, et al., 2018). RFP (mCherry, 15–40 min half maturation time)‐labeled *P. agricolaris* B1qs70 was used in this experiment, and the red fluorescent intensity was utilized as a measure of bacterial survivability (Figure 4b). The fluorescent intensities of free‐living bacteria and intracellular bacteria (within spores) were measured every 24 hours in the first week and every week after that up to 21 days in a light incubator (21°C, 60% light intensity). For each test, we dilute the selected bacteria (2 × 10^4^/mL) or spores (2 × 10^6^/mL) to the same concentration using KK2 buffer, and 200 μL bacterial or spore suspensions were measured. The same amount of spores without any *P. agricolaris* B1qs70 was measured to determine the autofluorescence of the spores. A microplate analyzer (Thermo, Varioskan LUX) measured the fluorescence intensity with 555 nm excitation wavelength and 585 nm emission wavelength (Merzlyak et al., 2007; Shaner et al., 2004). The average fluorescence intensity of the amoeba spores was 0.088, which was subtracted from the final analyses.

### 2.7 Statistical analyses

The stress effect, amoeba effect, and the stress×amoeba interaction effect were investigated on the survival of free‐living and intracellular bacteria using general linear models with Identity link in GraphPad Prism 9.0.0, followed by a post hoc Tukey test. A significant stress main effect indicates that stress can affect bacteria's fitness. A significant amoeba main effect indicates that the presence of amoeba can affect bacteria's fitness. A significant stress × amoeba interaction will indicate that bacteria have a higher fitness within amoeba in response to different environmental stressors.

## 3 RESULTS

### 3.1 Bacteria within spores have higher survival rates under environmental stress

**FIGURE 2:**
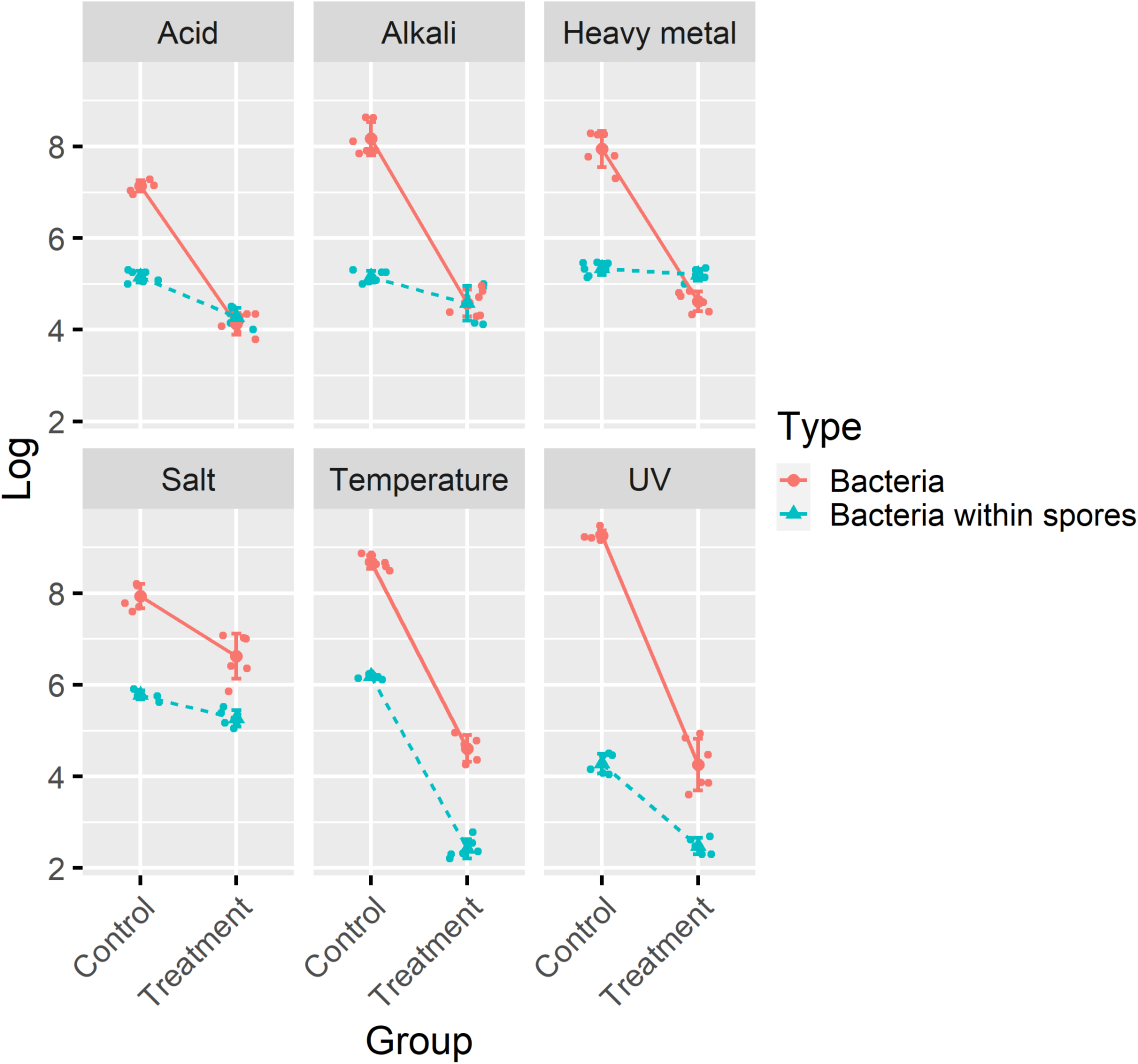
Survival of free‐living and intracellular bacteria in response to six environmental stressors. Control: the number of viable bacteria (free‐living and intracellular) without stress. Treatment: the number of viable bacteria (free‐living and intracellular) after exposure to different environmental stressors.

The exposure experiments showed that both environmental pollutants and amoeba could substantially affect bacteria's survival (Figure 2). All six environmental stressors significantly decreased free‐living bacteria's survival, as indicated by the significant main effects (Table 1). UV stress showed the strongest inactivation rate (−4.99 ± 0.51 log), while salt stress caused the lowest inactivation rate (−1.31 ± 0.24 log). However, bacteria within spores had significantly higher survival rates under environmental stress except for temperature, ranging from −0.13 ± 0.05 log to −3.76 ± 0.25 log.

**TABLE 1:** General linear models of the facultative bacteria in response to six environmental stressors.

	Type III SS	*F*	*p*
Heat stress			
Stress	87.69	87.69	**<.0001**
Amoeba	31.38	31.38	**<.0001**
Stress × Amoeba	0.1326	3.675	**.0704**
Salt stress			
Stress	4.989	55.99	**<.0001**
Amoeba	18.52	207.9	**<.0001**
Stress × Amoeba	0.9634	10.81	**.0037**
Alkali stress			
Stress	26.08	276.9	**<.0001**
Amoeba	13.66	145	**<.0001**
Stress × Amoeba	13.63	144.7	**<.0001**
Acid stress			
Stress	22.71	722.9	**<.0001**
Amoeba	5.063	161.1	**<.0001**
Stress × Amoeba	6.822	217.1	**<.0001**
Heavy metal			
Stress	18.04	303.8	**<.0001**
Amoeba	6.175	104	**<.0001**
Stress × Amoeba	15.26	257.1	**<.0001**
UV stress			
Stress	66.07	625.8	**<.0001**
Amoeba	65.31	618.6	**<.0001**
Stress × Amoeba	14.59	138.2	**<.0001**

*Note*: Significant effects are highlighted in bold.

Abbreviation: Type III SS, type III sum of squares.

General linear models showed significant stress × amoeba interactions under all environmental stressors except heat stress, indicating that bacteria have a higher fitness within the amoeba spore in response to different environmental pollution (Table 1, Figure 2). Amoeba also showed a high degree of protection over the UV stress, in which bacteria had a significantly higher survival rate (−1.75 ± 0.38 log) than free‐living bacteria (−4.99 ± 0.51 log). No significant protective effect was observed in response to heat stress (Stress × Amoeba, *p* = .07). The results of the normalized data were consistent with the above analyses (Figure S2, Table S1). Taken together, these results show that bacteria within spores have higher survival rates under various environmental stressors.

### 3.2 Morphology and survival of amoeba spores under environmental stressors

**FIGURE 3:**
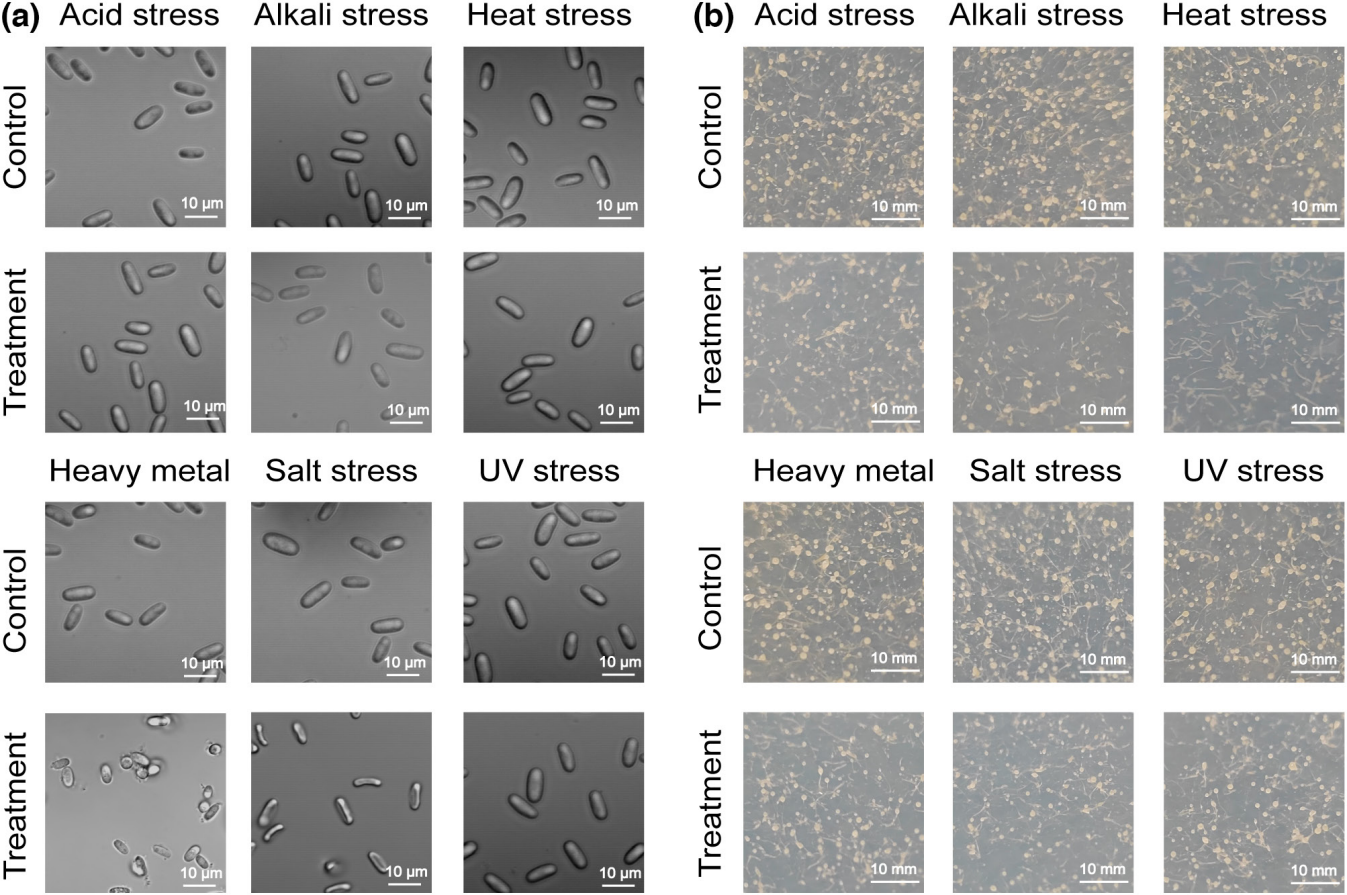
Morphology (a) and survival (b) of amoeba spores under environmental stressors. Scale: (a) 10 μm); (b) 10 mm.

We explore the possible mechanisms of amoeba protection using microscopy‐based morphological analyses and viability tests. We picked up amoeba spores by inoculation ring and observed the structural changes of the spores, and all amoeba spore cells remained intact under various environmental stressors (Figure 3a). No broken spores were observed, indicating they were highly stress tolerant. These extreme environmental stressors could not destroy their cell walls or membranes, which could explain why bacteria within spores have higher survival rates. Heavy metal and salt stress significantly changed the amoeba spore's appearance. Under heavy metal stress, spore cells became more transparent and aggregated together, indicating heavy metal had substantial interactions with the cell wall of amoeba spores (Figure 3a). Under salt stress, the spores gradually dehydrated and shrunk due to osmotic pressure (Figure 3a). Still, despite the apparent morphological change in the amoeba spore, bacteria within them had very high survival and were barely affected by the extreme extracellular environmental stress (Figure 2). In addition, we tested if amoeba spores were viable after treatments. The results showed that treated amoeba spores could still grow on bacterial lawns and form fruiting bodies within 5 days, indicating they were highly stress tolerant (Figure 3b). Of which, the heat stress treatment group grew significantly slower than other groups (Figure 3b).

### 3.3 Bacteria survive longer within amoeba spores than free living

**FIGURE 4:**
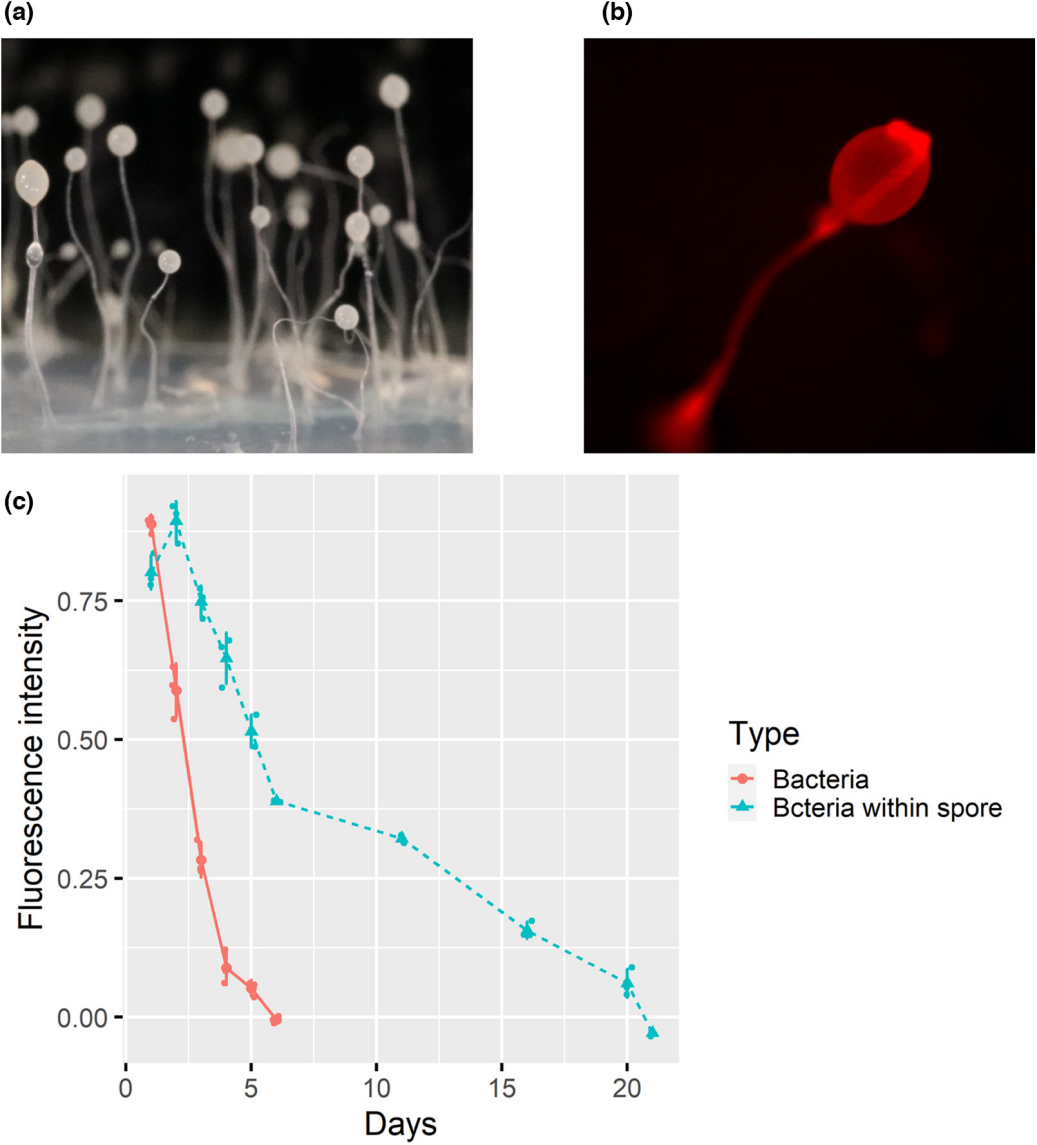
Comparison of bacterial survival between free‐living and intracellular bacteria. (a) Amoeba fruiting bodies; (b) Amoeba fruiting‐body bearing the red fluorescently labeled B1qs70 bacteria under a red fluorescence microscope; (c) Fluorescence intensity curves of free‐living B1qs70 and bacteria within spores as a function of time.

To further explore the potential benefit of being facultative symbionts, we compared the maximum survival time of free‐living bacteria versus intracellular bacteria within amoeba. The fruiting body of *D. discoideum* consisted of a stalk and a sorus on the top (Figure 4a). Figure 4b shows the distribution of *P. agricolaris* B1qs70 in the fruiting body, indicating that these symbionts are distributed in the spores and the stalks. Free‐living *Paraburkholderia* bacteria gradually died over time, and the fluorescent intensity declined from 0.88 to 0 within 6 days (Figure 4c). However, bacteria within amoeba spores showed a distinct survival pattern. First, fluorescent intensity significantly (*p* < .001) increased from 0.82 ± 0.03 to 0.91 ± 0.04 within 1 day (Figure 4c), indicating that *Paraburkholderia* bacteria could grow within amoeba spores. After that, the fluorescent intensity decreased with time and reached 0 at day 21 (Figure 4c), indicating that bacteria within spores could survive significantly longer than free‐living ones (6 days). These results show that living within amoeba gives bacteria a significant advantage over free‐living ones when environmental nutrition is limited.

## 4 DISCUSSION

### 4.1 Facultative symbionts benefit from living within amoeba hosts under environmental stress

Facultative symbionts are often acquired from the environment and can live independently, but it is not clear why they tend to live with amoeba hosts when other habitats are optional. Facultative symbionts are thought to benefit from their hosts, but this is often overlooked due to the difficulty of measuring symbiont fitness (Garcia & Gerardo, 2014). This study provides empirical evidence that facultative symbionts have higher survival rates under various environmental stressors by using a facultative amoeba–bacteria system. Facultative symbionts also survive significantly longer (more than three times) when food is limited in the environment. A previous study also showed that amoeba could protect *Lactobacillus pneumophilus* from the disinfection effects of chlorine and chlorine dioxide to a certain extent (Dupuy et al., 2011). Therefore, amoeba hosts may provide significant fitness advantages to their facultative symbionts.

Furthermore, this study shows that environmental stressors promote the persistence of facultative bacterial symbionts in amoebae and may favor the formation of symbiotic relationships at evolutionary scales. Human‐induced environmental pollution has severely impacted the diversity of life on Earth (Baker et al., 2018; Delgado‐Baquerizo et al., 2020; Shu et al., 2020). While most studies have focused on the effects of pollutants on individual species, how environmental stress affects the status of host–microbe interactions has been largely ignored (Benard et al., 2020; Wu et al., 2022). In this study, exposure experiments showed that both environmental pollutants and amoebae could significantly affect bacterial survival and population dynamics. Bacteria in amoeba hosts have significantly higher survival rates than free‐living bacteria that are nearly eliminated by environmental stressors. Such strong selection forces inevitably favor the formation of amoeba–bacteria symbioses, which promotes the persistence of facultative bacterial symbionts in amoebae. In addition, this study also shows that environmental stressors could favor the formation of symbiotic relationships at evolutionary scales. It has recently gained attention in symbiosis with a potential role for bet‐hedging to evade the long‐term effects of stress (Scott et al., 2022; Veresoglou et al., 2022). Therefore, we hypothesize that more facultative symbionts will be found in polluted environments than in natural environments, which should be studied in more detail in the future.

### 4.2 Environmental implications: Pollution and disinfection may increase the biosafety risk related to amoebae and their intracellular bacteria

This study has two significant environmental implications. First, environmental pollution may potentially select and produce more amoeba‐resisting bacteria. During the long‐term interactions between amoebae and bacteria, some bacteria have evolved to resist amoeba predation or even infect amoebae, which are called amoeba‐resisting bacteria (Shi et al., 2021). Because of the physiological similarity between amoebae and human phagocytic cells, many amoeba‐resisting bacteria are also human pathogens, such as *L. pneumophila*, *Mycobacterium,* and *Chlamydiae*, posing significant health risks (Butler et al., 2020; Shi et al., 2021; Strassmann & Shu, 2017). Therefore, environmental stress directly favors the formation of amoeba‐resisting bacteria and consequently produces potential pathogens. Although no study has explicitly tested such a hypothesis, some studies showed that genetic change induced by amoeba–bacterium interactions was plausible. For example, one study showed that multiple amoeba pathogenic virulence genes evolved during the interactions between bacteria and amoebae and identified the controlling genes (*SdhA*, *RavY*, etc.) of *L. pneumophila* in various amoeba (Park et al., 2020). Stallforth et al. (2013) found that a single derivation point mutation, which changed the metabolic pathway and products of the bacteria, led to the transformation of the inedible *Pseudomonas fluorescens* strain into an edible strain. Therefore, genetically speaking, environmental stress can potentially select and produce more amoeba‐resisting bacteria. However, this study only investigated one amoeba host, and future research should test more hosts.

Second, this study also provides significant implications for drinking water safety. Drinking water disinfection techniques, such as Cl_2_, ClO_2_, and UV, can effectively inactivate various bacteria in the drinking water system (Proctor & Hammes, 2015). However, they are less effective toward protozoa, including *Cryptosporidium*, *Giardia,* and amoebae (He et al., 2021). As a result, amoebae have been found in many drinking water systems, and they could resist exposure to 100 mg/L chlorine for 10 min (Storey et al., 2004). Therefore, the drinking water systems provide a very strong selective environment, promoting the formation of amoeba‐resisting bacteria. Indeed, a recent study provided direct evidence that amoeba spores could protect their intracellular bacteria from disinfection treatment (He et al., 2021). Given that drinking water is directly linked to human health, we believe it is necessary to disentangle the complex interactions between amoebae and their intracellular bacteria in the drinking water systems (Gomes et al., 2020). Future studies should investigate whether and how different drinking water disinfection techniques influence amoeba–bacterium interactions and the potential consequences for public health.

## AUTHOR CONTRIBUTIONS

**Zihe Wang:** Data curation (lead); formal analysis (lead); methodology (lead); visualization (equal); writing – original draft (equal). **Wei Huang:** Data curation (equal); investigation (equal); methodology (equal); writing – review and editing (equal). **Yingwen Mai:** Data curation (equal); formal analysis (equal); methodology (equal). **Yuehui Tian:** Formal analysis (equal); investigation (equal); methodology (equal); writing – review and editing (equal). **Bo Wu:** Data curation (equal); methodology (equal); visualization (equal); writing – review and editing (equal). **Cheng Wang:** Data curation (equal); investigation (equal); methodology (equal); visualization (equal); writing – review and editing (equal). **Qingyun Yan:** Formal analysis (equal); visualization (equal); writing – review and editing (equal). **Zhili He:** Investigation (equal); methodology (equal); writing – review and editing (equal). **Longfei Shu:** Conceptualization (lead); funding acquisition (lead); methodology (lead); project administration (lead); supervision (lead); writing – review and editing (lead).

## CONFLICT OF INTEREST STATEMENT

The authors declare no conflicts of interest.

## Supporting information


Appendix S1

## Data Availability

All data are available from the ScienceDB: https://www.scidb.cn/en/s/qUNnUj.
