# Supplementary material for: Environmental stress promotes the persistence of facultative bacterial symbionts in amoebae
Source: Ecol Evol. 2023 Mar 16;13(3):e9899. doi: 10.1002/ece3.9899 (PMC10019945; doi:10.1002/ece3.9899)
Supplement: Supplementary file 1 — Appendix S1 [file ECE3-13-e9899-s001.docx]

*Supporting Information for*

**Environmental stress promotes the persistence of facultative bacterial symbionts in amoebae**

Zihe Wang, Wei Huang, Yingwen Mai, Yuehui Tian, Bo Wu, Cheng Wang, Qingyun Yan, Zhili He, Longfei Shu*

*School of Environmental Science and Engineering, Southern Marine Science and Engineering Guangdong Laboratory (Zhuhai), Guangdong Provincial Key Laboratory of Environmental Pollution Control and Remediation Technology, Sun Yat-sen University, Guangzhou 510006, China*

***Correspondence**

Prof. Longfei Shu, [shulf@mail.sysu.edu.cn](mailto:shulf@mail.sysu.edu.cn)

School of Environmental Science and Engineering

Sun Yat-sen University

Guangzhou 510275, China

**This supporting information includes 4 pages (2 Figures and 1 Table).**


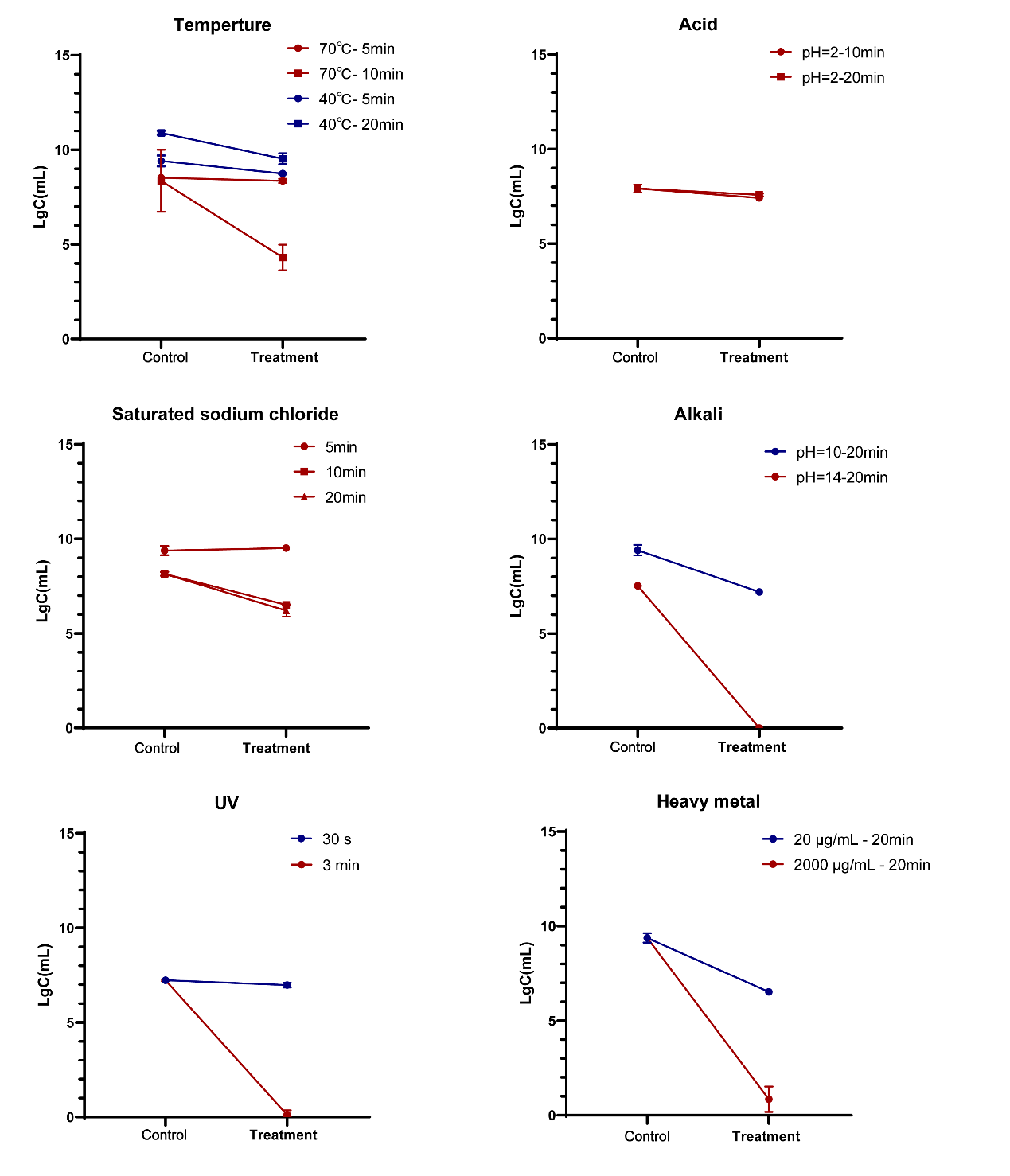


**Figure S1.** Preliminary experiments of bacteria survival under different environmental stressors.


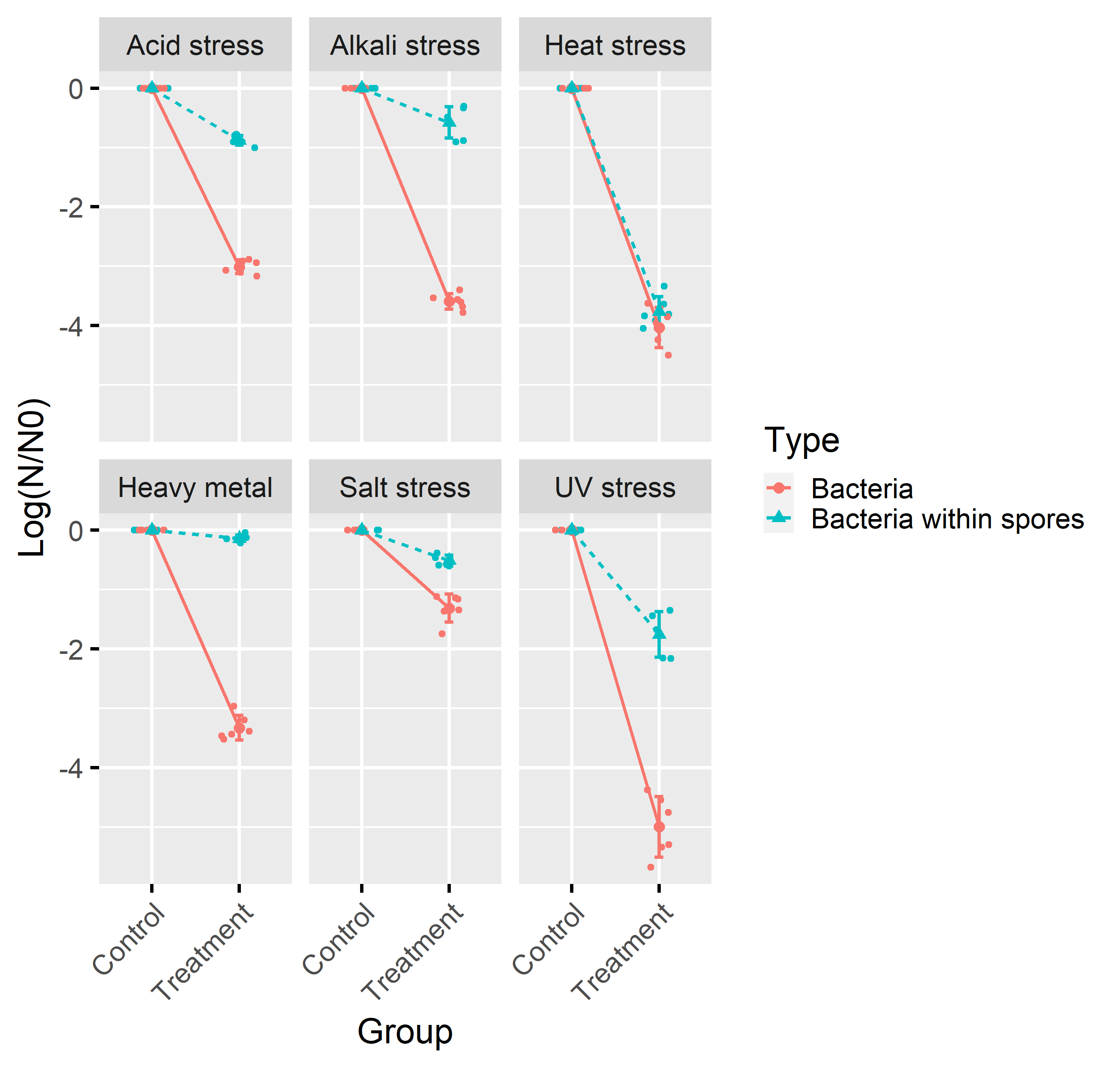


**Figure S2.** Survival of free-living and intracellular bacteria (normalized) in response to six environmental stressors.

**Table S1.** General linear models of the facultative bacteria (normalized) in response to six environmental stressors. Significant effects are highlighted in bold. Type III SS: type III sum of squares.

|  | **Heat Stress** | | |
| --- | --- | --- | --- |
|  | Type III SS | *F* | *P* |
| Stress | 86.93 | 2125 | **<0.0001** |
| Amoeba | 0.1044 | 2.552 | 0.1267 |
| Stress × Amoeba | 0.1044 | 2.552 | 0.1267 |
|  | **Salt Stress** | | |
|  | Type III SS | *F* | *P* |
| Stress | 4.989 | 310.5 | **<0.0001** |
| Amoeba | 0.9634 | 59.95 | **<0.0001** |
| Stress × Amoeba | 0.9634 | 59.95 | **<0.0001** |
|  | **Alkali Stress** | | |
|  | Type III SS | *F* | *P* |
| Stress | 26.08 | 1219 | **<0.0001** |
| Amoeba | 13.63 | 636.7 | **<0.0001** |
| Stress × Amoeba | 13.63 | 636.7 | **<0.0001** |
|  | **Acid Stress** | | |
|  | Type III SS | *F* | *P* |
| Stress | 22.71 | 4510 | **<0.0001** |
| Amoeba | 6.822 | 1354 | **<0.0001** |
| Stress × Amoeba | 6.822 | 1354 | **<0.0001** |
|  | **Heavy Metal** | | |
|  | Type III SS | *F* | *P* |
| Stress | 18.04 | 1517 | **<0.0001** |
| Amoeba | 15.26 | 1284 | **<0.0001** |
| Stress × Amoeba | 15.26 | 1284 | **<0.0001** |
|  | **UV Stress** | | |
|  | Type III SS | *F* | *P* |
| Stress | 63.05 | 305.3 | **<0.0001** |
| Amoeba | 18.51 | 89.65 | **<0.0001** |
| Stress × Amoeba | 18.51 | 89.65 | **<0.0001** |
